# Effect of *Alliaceae* Extract Supplementation on Performance and Intestinal Microbiota of Growing-Finishing Pig

**DOI:** 10.3390/ani10091557

**Published:** 2020-09-02

**Authors:** Cristian Jesús Sánchez, Silvia Martínez-Miró, Juan José Ariza, Josefa Madrid, Juan Orengo, María Arántzazu Aguinaga, Alberto Baños, Fuensanta Hernández

**Affiliations:** 1 Department of Animal Production, Faculty of Veterinary, Campus de Espinardo, University of Murcia, 30100 Murcia, Spain; cristianjesus.sanchez@um.es (C.J.S.); alimen@um.es (J.M.); jorengo@um.es (J.O.); nutri@um.es (F.H.); 2 DMC Research Center, Camino de Jayena, 82, 18620 Alhendín, Granada, Spain; jariza@dmcrc.com (J.J.A.); arancha.aguinaga@domca.com (M.A.A.); abarjona@domca.com (A.B.)

**Keywords:** *Allium* spp. extract, growing-finishing pigs, growth performances, intestinal microbiota, short-chain fatty acid profile, antioxidant capacity

## Abstract

**Simple Summary:**

The increasing interest in phytogenics for use with livestock, especially swine and poultry, is mainly due to their antimicrobial, antioxidant, growth-promoting, and gut microbiome modulation properties, which makes them ideal candidates to mitigate the negative effects of the ban on antibiotic growth promoters in the European Union. We tested the ability of *Allium* spp. extract (containing garlic and onion), one of the best-known phytogenics, used in pig feed, to improve growth performance through modulation of the microbiome and changes in the metabolism of short-chain fatty acids in the gut tract. The promising results obtained in the present study suggested that *Allium* spp. extracts had the potential to be used in feeding pigs to improve growth performances by modulating the microbiota and metabolism of short-chain fatty acids.

**Abstract:**

The aim of the present study was to ascertain whether an *Allium* spp. extract rich in organosulfur compounds, such as propyl thiosulfonate (PTSO), added to the feed of growing-finishing pigs at 5 g/kg enhances growth performance or affects the fecal microbiome, the levels of short-chain fatty acids, or the antioxidant capacity of the animals. Fifty male growing pigs (large white) of 23.07 ± 2.87 kg average body weight were randomly allotted to two treatments in a 103-day trial. The trial was divided into two periods, an initial growing phase (56-days) and a finishing phase (47-days). Two dietary treatments for each phase (growing and finishing) were used: a control diet (CON) and an experimental diet consisting of the control diet to which 5 g/kg of *Allium* spp. extract was added to substitute sepiolite (GAR). Throughout the study, body weight, average daily gain (kg/day, ADG), feed intake (kg/day), and feed conversion ratio (kg/kg) were measured, while the backfat thickness and muscle depth were determined at the end of the study. Besides, feces samples were taken for bacterial counts by means of real-time PCR and short-chain fatty acid (SCFA) profile determination, and the antioxidant capacity was assessed in serum and saliva. In the animals receiving *Allium* spp. extract (5 g/kg) in the feed, ADG increased (*p* < 0.05) throughout the trial, *Salmonella* spp. and *Clostridium* spp. counts in feces had decreased (*p* < 0.05) when measured on day 56, and, by day 103, *Salmonella* spp., *Clostridium* spp., and *Enterobacteriaceae* counts had decreased (*p* < 0.05) and *Lactobacillus* spp. counts had increased (*p* < 0.01) in feces. Regarding the SCFA profile in feces and antioxidant capacity measured in serum and saliva, supplementation with *Allium* spp. extract significantly increased the levels of propionic, isobutyric, and isovaleric acids and the percentage of total branched fatty acids, while the c2/c3 and (c2 + c4)/c3 ratios were lower (*p* < 0.05) in feces; the Trolox equivalent antioxidant capacity and the cupric reducing antioxidant capacity levels in serum were significantly higher in the same pigs on day 103 than on day 0. Consequently, based on the current results, *Allium* spp. extract rich in organosulfur compounds, added to the diet at 5 g/kg, had a beneficial effect on the microbiota and would seem to be a possible alternative for increasing the growth performance of growing-finishing pigs. However, further studies on the effects of *Allium* spp. supplementation on carcass quality are necessary.

## 1. Introduction

Since January 2006, the use of antibiotic growth promoters (AGP) for livestock has been banned in the European Union (Regulation 1831/2003/EC). The appearance of new antibiotic-resistant bacteria and the prohibition of antibiotics, such as AGP, have increased the need to develop new alternatives to antibiotics and growth promoters [1]. Numerous substances have been proposed for as feed additives: acidifiers (organic, inorganic, blends, or salts of acids); minerals, especially zinc, whether in its inorganic form, ZnO, (whose use at pharmacological levels will be banned from 2022), or in its organic form (Zn-methionine); prebiotics; probiotics; nucleotides; plant extracts from *Allium sativum* or *Origanum vulgare*, for example [2].

The use of phytogenic feed additives, such as garlic and onion, has gained increasing attention, especially for use in swine and poultry, for its antioxidant action, specific impact on dietary palatability and gut functions, antimicrobial actions, or growth-promoting efficacy [3].

Numerous compounds present in garlic or onion extract, such as allicin, alliin, phenols, or flavonoids, have demonstrated their antioxidant action by their free radical scavenging activity against 2,2-diphenyl-1-picrylhydrazyl radical (DPPH) and 2,2′-azino-bis-(3-ethylbenzo-thiazoline-6-sulfonic acid) diammonium salt (ABTS+) or their Fe (III) reducing ability (FRAP) in in vitro trials [4,5,6,7]. The antioxidative effects of garlic and onion have also been evaluated in in vivo trials; for example, Batcioglu et al. [8] reported a protective effect against radiotherapy in rats based on the antioxidant activity of garlic extract, and Vezza et al. [9] showed an anti-inflammatory activity that could be associated with the immunomodulatory properties of garlic and onion in mice. Other species, such as rabbit, have also been the target of studies on the reduction of oxidative toxicity, which, it has been proposed, maybe due to the high total phenolic content of garlic extract [10]. Even some human illnesses related to oxidative stress, such as rheumatoid arthritis or obesity, have been seen to improve as the result of taking garlic and onion supplements [11,12].

Probably the most promising route to improving host health in livestock using *Allium* spp. derivatives is through the dietary modulation of the intestinal microbiome. Thus, the effect of some organosulfurate compounds present in *Alliaceae*, such as propyl propane thiosulfonate (PTSO) or propyl propane thiosulfinate (PTS), have demonstrated modulatory activity both on beneficial bacteria (*Bifidobacterium* spp. or *Lactobacillus* spp.) and on pathogenic bacteria (*Escherichia coli*, *Salmonella typhimurium,* or *Clostridium* spp.) [13,14,15,16,17]. This study has found that some beneficial bacteria (e.g., *Lactobacillus* spp.) are more resistant to the antibiotic effect of garlic compounds than pathogenic bacteria like *Clostridium* spp. [17], and that there is a higher count of *Bifidobacterium* spp. in mucosa-associated microbiota of the ileum compared with the microbiota animals who are fed the control diet [14]. Because of its modulatory activity on gastrointestinal microbiota, *Allium* spp. derivatives emerge as promising alternatives for improving feed efficiency and growth performance in livestock. In this respect, Quan et al. [18] suggested that difference in microbiota composition in the different parts of the gut between pigs with a high feed conversion ratio (FCR) and those with a low FCR might confer on the former a greater ability to utilize dietary polysaccharides and proteins. Furthermore, the short-chain fatty acids (SCFAs) and indolic compounds produced by microbial fermentation might improve porcine feed efficiency and promote intestinal health. In the last 5 years, several studies have explored possible links between gut microbiota and feed efficiency or growth performance, especially in poultry and swine [19,20,21,22]. However, many of the mechanisms behind these correlations between microbiome composition and productive parameters remain unknown.

The antimicrobial and antioxidant activity shown by PTSO from *Allium* spp. extracts in animals could also have direct or indirect implications for the food industry. For example, antimicrobial activity against important pathogenic bacteria in food, such as *Salmonella enterica*, *Escherichia coli O157:H7*, *Clostridium perfringens*, *Listeria monocytogenes,* or *Campylobacter jejuni*, has been demonstrated when PTSO is used in the packaging [23,24]. Together with the use of organosulfurate compounds in active food packaging, the antimicrobial properties of these compounds could be useful for reducing foodborne zoonoses because of the way they act against bacteria, which can be transmitted from meat to consumers. In this regard, not many studies are available, but one of the most interesting studies has raised that rabbit diet supplemented with plant extract (onion) has improved the microbiological quality of the hind legs of slaughtered rabbits, although the results are limited [25]. In the case of pork, the second most widely consumed meat (kg/person/year) in the European Union [26], numerous biological agents are known to cause foodborne zoonoses in humans through its consumption. Among these agents, *Salmonella enterica*, *Clostridium botulinum,* and *Clostridium perfringens* [27] belong to bacterial genera against which garlic extract has been demonstrated as having an antimicrobial effect.

Given the ability of some organosulfur compounds present in garlic and onion, especially PTSO, to modulate the activity of some bacteria present in the pig’s digestive tract, it has been hypothesized that the administration of garlic and onion extract rich in PTSO to pigs in the growing-finishing period would enhance growth performance by improving the intestinal health of the animals. The present study aimed to evaluate whether supplementation of the diet with *Allium* spp. extract (garlic and onion) at 5 g/kg would improve the growth performances of growing-finishing pigs. The effects on fecal microbiota and short-chain fatty acids and the antioxidant status of the pigs were also analyzed.

## 2. Materials and Methods

### 2.1. Ethical Statement

This research was carried out at the installations of the Veterinary Teaching Farm of the University of Murcia, Spain, with the approval of the Animals Experimentation Ethics Committee of the University of Murcia (4 August 2017, n° A-13170805) and following the European Union guidelines for the care and use of research animals (Directive 2010/63/EU of the EU Parliament and of the Council of 22 September 2010 on the protection of animals used for scientific purposes).

### 2.2. Source of Allium spp. Extract

The *Allium* spp. extract (GARLICON^®^) used in the study was supplied by DOMCA Co., Granada, Spain. The product contained garlic and onion and was rich in propyl propane thiosulfonate (PTSO), an organosulfur compound characteristic of *Allium* species. GARLICON^®^ was added in the powder form to the animal feed at 5 g/kg (equivalent to 30 ppm of the active ingredient) [13].

### 2.3. Experimental Design, Animals, Facilities, and Diets

A total of fifty male growing pigs (large white), with 23.07 ± 2.87 kg of average body weight (BW), were randomly allotted to two treatments, using 5 pens per treatment and five pigs per pen, in a 103-day trial. The trial was divided into two periods, an initial growing phase (56-day trial; 23 to 60 kg BW average) and a finishing phase (47-day trial; 60 to 110 kg average BW). Two dietary treatments for each phase (growing and finishing) were followed: control diet (CON) and an experimental diet (GAR, control diet + 5 g/kg of *Allium* spp. extract to replace sepiolite). Both diets were formulated according to the Spanish Foundation for the Development of Animal Nutrition (Fundación Española para el Desarrollo de la Nutrition Animal, FEDNA) [28]. The feed used in each batch was sampled to analyze the chemical composition (Table 1).

During the growing and finishing period, animals received dietary treatments for 56 days and 47 days, respectively. All pens were equipped with a steel nipple waterer and a plastic feeder with ad libitum access to water and feed. Incidents and/or deaths were monitored throughout the experiment.

### 2.4. Growth Performance and Sampling

The body weight of each animal was recorded at days 0, 56 (end of growing period), and 103 (end of finishing period), and the daily feed intake (FI) per replicate was monitored. The average daily gain (ADG) and feed conversion ratio (FCR) were calculated using the BW and FI data.

At the end of the finishing period, backfat thickness and muscle depth were measured in all the pigs by ultrasound scan with a linear probe (SF-1 Wireless Backfat and Loin Depth Scanner, Sonivet, Beijing, China), which is a B-mode ultrasound device equipped with a 45 mm, 5.0-MHz linear array, taking measurements 6.5 cm from the midline between the 13th and 14th last ribs to determine any changes in carcass characteristics.

At 0, 56, and 103 days, two animals per replicate were randomly selected, among those of average weight, to take fecal samples by stimulating-massaging the anus. For microbiota analysis, fecal samples were collected in sterile containers and refrigerated at 4 °C before they were delivered to the laboratory, and then frozen at −80 °C until analysis. In addition, feces from day 103 were collected for SCFA analysis, acidifying the subsamples (1 g/sample) with 0.032 mL H_2_SO_4_ (50:50) and storing at −20 °C until use.

On 0 and 103-day of the experimental period, serum and saliva samples were obtained from the same animals from whom the feces blood samples were collected via jugular venipuncture into 5 mL tubes without additives, centrifuged at 3000× *g* for 10 min, and the serums were stored at −80 °C until analysis. Saliva samples were taken using Salivette^®^ tubes (Sarstedt, Aktiengesellschaft & Co. D-51588 Nümbrecht Nümbrecht, Germany), containing a sponge instead of a cotton swab clipped to a thin flexible metal rod [29], which was kept in the pigs’ mouths for 1–2 min until thoroughly moist. The sponge was then placed in the tube and centrifuged at 3500× *g* for 10 min and stored frozen at −80 °C until analysis.

### 2.5. Feed, Serum, and Feces Analyses

Feed samples were ground to pass through a 1-mm mesh in an ultra-centrifugal mill (Retsch ZM 200, Retsch GmbH, Haan, Germany) and analyzed for dry matter (DM), ash, and crude protein (CP) content following the procedures of the Official Analytical Chemists Association [30]. The dry matter was determined by drying the feed samples at 103 °C to reach a constant weight (934.01 method), and the ash content of a weighed feed sample was determined in a muffle furnace (Hobersal HK-11, Barcelona, Spain) at 550 °C for 4 h. The crude protein content of the diets was analyzed by the Kjeldahl method (984.13 A-D; Kjeltec 8400 Analyzer Unit FOSS, Höganäs, Sweden). The CP data were expressed as total N × 6.25. The neutral detergent fiber content was analyzed using an ANKOM 220 fiber analyzer unit, according to Van Soest et al. [31]. For the mineral analysis, feed samples were ashed at 550 °C for 4 h in a muffle furnace. Ash samples were diluted in 0.6 N HNO_3_ solutions and filtered to analyze the P and Ca concentration. P was measured by the vanadate–molybdate method according to the official analytical method described in the Boletin Oficial del Estado [32], and the Ca was determined with an atomic absorption spectrophotometer (Unicam M series, Solaar House, Thermo Fisher Scentific, Waltham, MA, USA).

In addition, to ensure the concentration of PTSO in feeds, UHPLC-ESI-MS/MS analyses were performed, according to the method described by Abad et al. [33].

To measure the antioxidant levels in serum and saliva, three different spectrophotometric methods were used: cupric reducing antioxidant capacity (CUPRAC); ferric reducing ability of plasma (FRAP); Trolox equivalent antioxidant capacity (TEACH) [34]. In all cases, the resulting data were expressed as millimoles of Trolox equivalents per liter (mmol/L).

Short-chain fatty acids in feces were analyzed, according to Oliveira et al. [35].

### 2.6. Real-Time Quantitation PCR

DNA was extracted from feces samples using the Favor Prep Stool Isolation Mini kit (Favorgen, Vienna, Austria) following the manufacturer’s protocol. The quantity of DNA was analyzed spectrophotometrically (Qubit 4 Fluorometer; Invitrogen, Thermo Fisher Scientific, Waltham, MA, USA). For DNA amplification, the primers used are listed in Table 2. Real-time PCR (qPCR) quantification assays were conducted in 96-well polypropylene plates (Multiplate 96-well PCR Plates, Biorad Laboratories, Hercules, CA, USA) using thermocycler CFX96 Real-time PCR Detection System (Biorad Laboratories, Hercules, CA, USA). For qPCR DNA amplification, the total volume employed in each reaction was 50 µL, which contained 10 µL SYBR GREEN buffer (Sensifast SYBR & Fluorescein kit, Thermo Fisher Scientific), 0.5 µL of the polymerase, 1.5 µL of each primer, 31.5 µL of RNAse free water, and 5 µL of DNA extract. The amplification involved the following thermocycling conditions: one cycle at 50 °C for 2 min, followed by a denaturation cycle at 95 °C for 5 min, 39 cycles of 15 s denaturation at 95 °C + 1 min at 60 °C (annealing) + 30 s at 72 °C (extension). After amplification, a melting curve analysis was made from 55 °C to 95 °C, with increments of 0.5 °C every 10 s. For the quantification of the target DNA copy number, a standard curve was generated. The bacterial concentration in each sample was measured as a log10 copy number by interpolating the values obtained from the fecal samples into the standard calibration curves. Each plate included duplicate reactions per DNA sample and the appropriate set of standards.

### 2.7. Statistical Analyses

The DNA copy numbers were logarithmically transformed (log10 DNA copy number) to meet a normal distribution before statistical analysis. Data performance parameters’ (except bodyweight) microbiology and SCFA were analyzed by independent-samples t-test. The changes in body weight along time were assessed using a one-way analysis of variance of repeated measures. The serum and saliva measurement data were subjected to two-way ANOVA using treatment and animal age as fixed effects. In all cases, the IBM SPSS Statistics system (IBM Corporation, Armonk, NY, USA) was used. The experimental unit was the pen, except for body weight data, where the pig represented the experimental unit. In all statistical analyses, differences were taken to be significant at *p* ≤ 0.05, while 0.05 < *p* ≤ 0.10 were considered near-significant trends.

## 3. Results

The average concentration of PTSO in feeds (32 mg/kg of DM) was as expected.

### 3.1. Growth Performances

The effects of dietary treatment on body weight and growth performance are shown in Table 3 and Table 4, respectively. The addition of *Allium* spp. extract (30 ppm PTSO) to feed increased 0–103 d ADG (*p* = 0.042), and body weight tended to show an increase when measured at the end of the finishing period (*p* < 0.10) compared with animals receiving the control diet. At 103 d, the dietary treatment was seen not to have affected (*p* > 0.05) backfat thickness or loin thickness in either group.

### 3.2. Fecal Microbiota

At the beginning of the trial (day 0), no significant differences in bacterial counts were found between the pigs assigned to each treatment. At day 56 (end of growing period), pigs fed the GAR diet showed a lower (*p* < 0.05) concentration of *Salmonella* spp. and *Clostridium* spp. in feces than the pigs fed the control diet (Table 5). The pigs receiving the garlic extract diet also showed a trend (*p* < 0.10) towards a lower concentration of *Enterobacteriaceae* in feces and a higher concentration of *Lactobacillus* spp. than the animals receiving the control diet. No differences (*p* > 0.05) in *Bifidobacterium* spp. concentration were found in feces between the dietary treatments.

At day 103 (end of finishing period), pigs fed the GAR diet showed a lower (*p* < 0.05) concentration of *Salmonella* spp., *Clostridium* spp., and *Enterobacteriaceae* and a higher concentration of *Lactobacillus* spp. (*p* < 0.001) in feces compared with pigs fed the CON diet. The concentration of *Bifidobacterium* spp. tended (*p* < 0.10) to be higher in pigs receiving GAR.

### 3.3. Short-Chain Fatty Acids Profile in Feces

The total content of volatile fatty acids and molar proportions of acetic, propionic, butyric, isobutyric, and isovaleric acids in the feces of pigs at the end of the trial are shown in Table 6. Supplementation of the diet with *Allium* spp. extract increased propionic, isobutyric, isovaleric acid and total ramification fatty acid percentages (*p* < 0.05) in feces. Animals fed the *Allium* spp. extract diet also showed a trend to increase total volatile fatty acids (*p* < 0.10) and to decrease acetic acid (*p* < 0.10) percentages in feces. The c2/c3 and (c2 + c4)/c3 ratios were lower (*p* < 0.05) in the feces of pigs fed the GAR diet.

### 3.4. Antioxidant Capacity in Serum and Saliva

The effects of dietary *Allium* spp. extract on the antioxidant capacity in serum and saliva are shown in Table 7. No differences (*p* > 0.05) were found at day 0 or day 103 in TEACH, FRAP, and CUPRAC activity in the serum and saliva of the pigs receiving either diet. In terms of sample time, the TEACH and CUPRAC levels were higher (*p* < 0.05) in the serum of pigs at day 103 compared with the same pigs at day 0. No interaction between time and treatment was observed for any parameter studied.

## 4. Discussion

The EU-wide prohibition on the use of antibiotic growth promoters in recent years (1831/2003/EC, 2006) and the 2022 EU objective to prohibit ZnO in animal feed have led to fresh research into alternative feed additives for pig feed, such as plant extracts, prebiotics, probiotics, yeasts, and nucleotides [2]. The results of the present study into the use of *Allium* spp. extract as animal feed additive suggested that the inclusion of this product at a level of 30 ppm of PTSO in the growing-finishing pig diets could improve growth performance parameters. For example, the ADG of pigs receiving the extract increased by up to 7%, which could be due to the PTSO effect or any other compound present in *Allium* spp. extract. This observation agreed with those of Samolińska et al. [43], who obtained similar results for ADG (0.900 kg/day) in growing-finishing pigs (from 25 to 115 kg) fed a diet that included 5 g/kg lyophilized garlic compared with the 0.852 kg/day gain measured in animals receiving a control diet. However, there is no consensus about the effects of garlic extract on some performance parameters in growing-finishing pigs. For example, supplementation with garlic extract at 1 or 10 g/kg of feed in pigs of 42 kg BW until slaughter did not affect ADG, while it had a significantly lower FI and higher FCR compared with pigs offered a control diet [44].

*Allium* spp. Extract, used as a feed additive, probably affects ADG and other growth performance parameters in livestock by increasing nutrient digestibility as a result of improvements in the gut microenvironment, enhanced pancreatic enzyme activities, and even increased villus height and mucosal thickness [15,16,45]. In the case of poultry, *Allium* spp. derivatives have been reported to positively affect growth performances as a consequence of organosulfur compounds, influencing the gut nutrient absorption and maintaining an optimal gut microbiome [46]. The current study found no differences in FI, despite that some findings have reported a significant decrease in pigs fed with dietary garlic owing to lower FI affecting FCR [44]. This reduction in FI could be due to the organoleptic properties of garlic, which would affect pigs’ feed intake, due to the importance of taste and smell in these animals [47]. No such reduction in FI due to the organoleptic properties of *Allium* derivate was observed in the current study. In fact, numerically higher feed intake was observed in this research, reflecting very precisely the increase in average daily gain if FCR is taken into account.

No differences in backfat thickness due to the diet received were observed. This agreed with Omojola et al. [48], who also observed no effect on the backfat thickness of 0.5% garlic extract supplementation, while there was no significant decrease when higher concentrations (1 and 1.5%) were administered.

By contrast, Grela et al. [49] showed that inulin and garlic extract added to the drinking water of pigs resulted in lower backfat thickness values compared with pigs receiving no such supplementation. The reason for these differences between studies could be due to the action of inulin or the garlic extract concentration tested or different ways of administration (in the water or feed) and perhaps even the format of the extract.

The capacity of *Allium* spp. organosulfur compounds, such as PTS and PTSO, to modulate the intestinal microbiota is widely known [13,15,16,50]. As regards the impact of the experimental diet on the fecal microbiota, the *Allium* spp. extract diet was associated with a lower *Salmonella* spp. and *Clostridium* spp. population in pig feces, both in growing and finishing animals, and a decrease in the *Enterobacteriaceae* population of finishing samples. These results support previous studies, which described the antimicrobial activity of PTSO and PTS against *Clostridium* spp. and *Enterobacteriaceae* in swine fecal microbiota in vitro and a bactericidal effect against *Salmonella* spp., as seen from time-kill curves [13]. Thiosulfinate compounds, such as PTS and PTSO, are produced by the decomposition of the principal thiosulfinate compounds, allicin and alliin, present in garlic and onion bulbs, whose main antimicrobial activity is due to the chemical reaction between these compounds and the thiol groups of acetate kinase and phosphotransacetyl present in bacteria [51]. Kim et al. [50] reported an association between a reduction in the quantity of fecal enterobacteria, such as *Escherichia coli* and *Clostridium perfringens,* and improved animal performance. However, as regards beneficial bacteria in pig feces, such as *Lactobacillus* spp., a lactic acid bacterium, our trial found a higher *Lactobacillus* spp. population in garlic-fed pigs than in their control counterparts. Such differential behavior in studies using *Allium* spp. extracts—reinforcing beneficial gut bacteria and inhibiting potentially harmful enterobacteria—has been documented before [52]. Why this occurs is not clear, but some authors propose the different composition of bacterial membranes, and hence their permeability, to allicin as an explanation [53]. In human medicine, PTS and PTSO have a significant broad-spectrum antibacterial activity against Gram-negative and Gram-positive multidrug-resistant bacteria isolated from human clinical samples [54].

The capacity of beneficial gut bacteria to adapt to the exposure of *Allium* spp. derivatives and the evidence that points to the antimicrobial activity against pathogenic bacteria through the organosulfur compounds it contains suggest this could be a good way to improve gut health in pigs and thus enhance growth performances. Although we do not know if there could also have other beneficial effects on the health of the pigs.

The short-chain fatty acids produced by the gut microbiome are mainly acetate, propionate, and butyrate, which have several functions in the intestinal tract as an energy source by the colonocytes, modulating host-microbiome interactions, in immune system maturation, in regulating inflammatory responses, defending the host against intestinal pathogens, and affecting gut homeostasis [55,56]. At the end of the finishing period, the present study found higher molar proportions of propionate in the feces of pigs receiving *Allium* spp. extract in their feed. It is well known that SCFA formation in the gut is regulated by many dietary, environmental, and microbiological factors, and that substrate availability, species composition of the microbiota, and intestinal transit time determine the amount and types of SCFA produced [57]. The higher molar proportion of propionate observed in the feces of GAR pigs than in those fed the CON diet may have been due to variations in the microbiome composition caused by *Alliaceae’s* active principles. At the end of the finishing period, *Lactobacillus* spp. counts in the feces of pigs fed *Allium* spp. extract was higher than in those fed the control diet. Previous experiments have demonstrated that a broiler diet supplemented with *Lactobacillus* strain had a higher concentration of lactic, propionic, and butyric acids in the caecum compared with other groups [58]. In addition, we also found an increase in branched-chain fatty acids in the feces pigs fed garlic. The branched SCFA is a result of the proteolytic metabolism and is produced through the degradation of branched-chain amino acids by intestinal microbiota but to a lesser extent than acetate, propionate, or butyrate [59]. However, increased proteolytic activity in the hindgut could generate other undesirable compounds, such as indole and skatole [60], which can generate sexual odor in the meat. Leong et al. [61] showed that dietary garlic led to higher skatole and indole concentrations in pork fat, though they attributed it to the influence of sulfur compounds (allyl di- and tri-sulfides) on the activity of certain hepatic enzymes involved in the hepatic metabolism of skatole. The results obtained in this study could also explain higher levels of skatole and indole due to greater proteolytic activity at the intestinal level. The antioxidant activity of garlic is based on its ability to increase the synthesis of endogenous antioxidant substances or reduce the production of oxidants, mainly oxygen-free radical species (ORS) [50]. Thus, garlic extract has been found to increase the activities of some antioxidant enzymes, such as superoxide dismutase, and decrease glutathione peroxidase in rats [62]. Supplementation of the pig diet with *Allium* spp. extract did not affect the antioxidant capacity of serum nor the antioxidant capacity of saliva. Few studies have investigated the improvements in the antioxidant capacity of the serum by *Allium* spp. in animals. In one study, Gorinstein et al. [63] reported a significant increase in the plasma ABTS+ (14.7%) and CUPRAC (13.9%) activities in rats fed a raw garlic-supplemented diet compared with rats fed a control diet without garlic.

## 5. Conclusions

The present study showed that the addition of *Allium* spp. extract at a level of 30 ppm of PTSO to the feed had beneficial effects on the growth performance of growing-finishing pigs. Moreover, *Allium* spp. dietary treatment produced a selective increase in certain beneficial bacteria (*Enterobacteriaceae and Lactobacillus* spp.) in the feces of pigs accompanied by a decrease in the numbers of pathological bacteria (*Clostridium* spp. and *Salmonella* spp.), as measured at the end of both periods, growing and finishing. Besides, the inclusion of garlic and onion extract in the diet modified the short-chain fatty acid profile, increasing the levels of propionic, isobutyric, and isovaleric acids and the percentage of total branched fatty acids of the pig feces. Finally, no effects of diet on the markers of antioxidant capacity of serum or saliva were noticed. In conclusion, the current study revealed that supplementation of the feed with organosulfur compounds from *Allium* spp. extract could be a possible alternative to improve growth performance by modulating the microbiota in growing-finishing pigs. However, further studies on the effects of *Allium* spp. supplementation on carcass and meat quality are necessary.

## Figures and Tables

**Table 1:** Diet Composition (%) and nutrient levels of diets (g/kg, as-fed basis) for growing-finishing pigs.

Raw Material	Growing Diets ^1^		Finishing Diets ^2^	
	CON ^3^	GAR	CON	GAR
Barley	74.57	74.57	10.74	10.74
Corn	-	-	41.19	41.19
Wheat	-	-	15.00	15.00
Soybean meal	17.72	17.72	15.55	15.55
Wheat bran	-	-	10.00	10.00
Lard	3.675	3.675	4.03	4.03
Calcium carbonate	0.824	0.824	1.11	1.11
Monocalcic phosphate	0.342	0.342	0.78	0.78
Salt	0.500	0.500	0.41	0.41
DL-Methionine	0.101	0.101	0.05	0.05
L-Lysine-HCl	0.330	0.330	0.28	0.28
L-Threonine	0.123	0.123	0.06	0.06
Dextrose	1	1		
GARLICON^® 4^	-	0.5	-	0.5
Sepiolite	0.5	-	0.5	-
Premix ^5^	0.3	0.3	0.30	0.30
Calculated nutrient composition (g/kg, as-fed basis) ^6^				
Crude protein (N × 6.25)	164.0	164.0	149.6	149.6
Ether extract	52.4	52.4	64.1	64.1
Neutral detergent fiber	151.8	151.8	138.6	138.6
Acid detergent fiber	50.5	50.5	46.2	46.2
Acid detergent lignin	6.0	6.1	8.1	9.9
Crude fiber	43.0	43.0	36.4	36.4
NE ^7^, Mcal/kg	2.45	2.45	2.40	2.40
SID ^8^ lysine	9.4	9.4	8.0	8.0
Analyzed nutrient composition (g/kg, as dry matter basis)				
Dry matter	908.0	912.0	910.0	909.0
Crude protein	176.8	177.0	159.2	158.9
Neutral detergent fiber	176.0	168.0	145.0	143.0
Ca	4.6	4.5	4.7	6.5
P (total)	3.0	3.1	3.9	4.0

^1^ From 0 to 56 d trial, ^2^ from 57 to 103 d trial. ^3^ Diet: CON, control; GAR, the inclusion of 5 g/kg of *Allium* spp. extract.^4^ *Allium* spp. Extract, rich in propyl propane thiosulfonate (PTSO), is marketed under the trademark Garlicon^®^ and was added to the feed in the powder form. ^5^ The premix provided the following vitamins and minerals (per g of diet): vitamin A, 6000 IU; vitamin D3, 450 IU; vitamin E, 15 IU; vitamin K3, 0.80 mg; biotin, 0.01 mg; vitamin B1, 1.0 mg; vitamin B2, 2.5 mg; vitamin B6, 1.5 mg; vitamin B12, 0.015 mg; nicotinic acid, 15 mg; pantothenic acid, 10 mg; choline chloride, 100 mg; Mn, 25 mg as MnO; Zinc, 90 mg as ZnO; I, 0.40 mg as KIO3; Cu, 20 mg as a cupric chelate of glycine hydrate; Se, 0.20 mg as Na2SeO3; Fe, 60 mg as FeCO3; 6-phytasa, 0.40 FTU; xylanase, 0.56 TXU; beta-glucanase, 250 TGU. ^6^ According to Spanish Foundation for the Development of Animal Nutrition (FEDNA, 2013). ^7^ Net energy. ^8^ Standardized ileal digestible lysine.

**Table 2:** Primer sequences used for the real-time PCR (qPCR).

Taxonomic Rank	Primer Sequence (5′-3′)	Product Length (bp)	Reference
*Enterobacteriaceae*	F: ATGGCTGTCGTCAGCTCGT	385	[36] ^1^
	R: CCTACTTCTTTTGCAACCCACTC		[37] ^1^
*Lactobacillus* spp.	F: AGCAGTAGGGAATCTTCCA	341	[38]
	R: CACCGCTACACATGGAG		
*Bifidobacterium* spp.	F: CGCGTCYGGTGTGAAAG	244	[39]
	R: CCCCACATCCAGCATCCA		
*Salmonella* spp.	F: TTGTTACGGCTATTTTGACCA	521	[40]
	R: CTGACTGCTACCTTGCTGATG		
*Clostridium* spp.	F: ATGCAAGTCGAGCGAGTG	122	[41]
	R: TATGCGGTATTAATCTCTCCTTT		

^1^ Modified by Castillo et al. [42].

**Table 3:** The effect of dietary inclusion of *Allium* spp. extract on body weight of growing-finishing pigs.

Groups	Initial	2 Weeks	4 Weeks	6 Weeks	8 Weeks	10 Weeks	12 Weeks	Final (103 d)	SEM ^2^	*p*-Value		
										Time	Diet	T × D ^3^
CON ^1^	23.0	30.8	41.2	52.8	64.2	79.5	98.8	120.4	1.029	0.000	0.172	0.079
GAR	23.2	31.5	42.4	54.2	67.4	84.3	103.7	127.1				

^1^ Diet: CON, control; GAR, the inclusion of 5 g/kg of *Allium* spp. extract (30 ppm PTSO). ^2^ Standard error of the mean (*n* = 25 replicates per treatment). ^3^ Time × Diet.

**Table 4:** The effect of dietary inclusion of *Allium* spp. extract on the in vivo performance of growing-finishing pigs.

Groups	CON ^1^	GAR	SED ^2^	*p*-Value
(0–56 d)				
Average daily gain (kg/day)	0.74	0.79	0.032	0.110
Feed intake (kg/day)	1.64	1.73	0.094	0.349
Feed conversion ratio (kg/kg)	2.20	2.16	0.045	0.427
(57–103 d)				
Average daily gain (kg/day)	1.19	1.27	0.048	0.122
Feed intake (kg/day)	2.85	3.07	0.122	0.119
Feed conversion ratio (kg/kg)	2.45	2.49	0.057	0.428
(0–103 d)				
Average daily gain (kg/day)	0.95	1.01	0.030	0.042
Feed intake (kg/day)	2.21	2.36	0.095	0.152
Feed conversion ratio (kg/kg)	2.34	2.35	0.040	0.804
Backfat thickness (mm)	11.4	12.2	0.294	0.146
Loin thickness (mm)	47.5	48.9	0.791	0.357

^1^ Diet: CON, control; GAR, the inclusion of 5 g/kg of *Allium* spp. extract (30 ppm PTSO). ^2^ Standard error of the differences (*n* = 5 replicates per treatment).

**Table 5:** The effect of dietary addition of *Allium* spp. extract on the intestinal contents of *Enterobacteriaceae*, *Lactobacillus* spp., *Bifidobacterium* spp., *Clostridium* spp., and *Salmonella* spp. (log10 number of copies DNA/g feces) in growing-finishing pigs.

Taxonomic Rank		CON ^1^	GAR	SED ^2^	*p*-Value
Initial (Day 0)					
Family	*Enterobacteriaceae*	6.69	6.59	0.174	0.595
Genus	*Lactobacillus* spp.	7.54	7.45	0.840	0.920
Genus	*Bifidobacterium* spp.	6.77	6.78	0.253	0.976
Genus	*Clostridium* spp.	8.73	9.00	0.428	0.546
Genus	*Salmonella* spp.	4.50	4.44	0.286	0.835
End of growing phase (day 56)					
Family	*Enterobacteriaceae*	6.39	5.77	0.300	0.053
Genus	*Lactobacillus* spp.	7.59	8.11	0.275	0.076
Genus	*Bifidobacterium* spp.	6.60	6.69	0.117	0.431
Genus	*Clostridium* spp.	8.65	7.76	0.346	0.019
Genus	*Salmonella* spp.	4.53	4.09	0.178	0.023
End of finishing phase (day 103)					
Family	*Enterobacteriaceae*	6.37	5.69	0.125	<0.001
Genus	*Lactobacillus* spp.	8.35	8.99	0.124	<0.001
Genus	*Bifidobacterium* spp.	6.53	6.84	0.152	0.058
Genus	*Clostridium* spp.	8.78	8.32	0.141	0.005
Genus	*Salmonella* spp.	4.63	4.25	0.109	0.003

^1^ Diet: CON, control; GAR, the inclusion of 5 g/kg of *Allium* spp. extract (30 ppm PTSO). ^2^ Standard error of the differences (*n* = 5 replicates per treatment).

**Table 6:** Total volatile fatty acids and molar percentage of volatile fatty acids in the feces of finishing pigs.

Item	CON ^1^	GAR	SED ^2^	*p*-Value
Total volatile fatty acids (mmol)	45.1	50.1	2.510	0.066
Acetic acid (%)	66.2	63.2	1.617	0.080
Propionic acid (%)	17.6	18.7	0.400	0.021
Isobutyric acid (%)	1.75	2.47	0.132	<0.001
Butyric acid (%)	9.78	9.65	1.121	0.912
Isovaleric acid (%)	1.98	3.06	0.190	<0.001
Valeric acid (%)	2.65	2.99	0.192	0.101
c2 ^3^/c3 ^4^	3.77	3.39	0.152	0.027
(c2 + c4 ^5^)/c3	4.32	3.91	0.132	0.007
Total branched fatty acids (%)	3.73	5.53	0.320	<0.001

^1^ Diet: CON, control; GAR, the inclusion of 5 g/kg of *Allium* spp. extract (30 ppm PTSO). ^2^ Standard error of the differences (*n* = 5 replicates per treatment). ^3,4,5^ Acetic acid, propionic acid, and butyric acid, respectively.

**Table 7:** The effect of sampling time and dietary treatment on serum and saliva antioxidant capacity in pigs.

Item	Day 0 (23 kg BW)		Day 103 (120 kg BW)			*p*-Value		
	CON ^1^	GAR	CON	GAR	SEM ^2^	Time	Treatment	T × T ^6^
Serum								
TEACH ^3^ (mmol/L)	0.41	0.39	0.47	0.49	0.012	0.007	0.996	0.388
FRAP ^4^ (mmol/L)	0.37	0.35	0.42	0.44	0.020	0.110	0.910	0.586
CUPRAC ^5^ (mmol/L)	0.32	0.32	0.37	0.38	0.009	0.009	0.876	0.528
Saliva								
TEACH (mmol/L)	0.65	0.63	1.04	0.93	0.102	0.118	0.832	0.795
FRAP (mmol/L)	1.25	1.25	1.49	1.36	0.200	0.553	0.951	0.818
CUPRAC (mmol/L)	0.57	0.57	0.73	0.71	0.079	0.335	0.989	0.877

^1^ Diet: CON, control; GAR, the inclusion of 5 g/kg of *Allium* spp. extract (30 ppm PTSO). ^2^ Standard error of the mean (*n* = 5 replicates per treatment). ^3,4,5^ Trolox equivalent antioxidant capacity, ferric reducing ability of plasma, and cupric reducing antioxidant capacity, respectively.^6^ Time × Treatment.

## References

[1] Upadhaya S.D., Kim I.H. (2017). Efficacy of phytogenic feed additive on performance, production and health status of monogastric animals—A review. Ann. Anim. Sci..

[2] Liu Y., Espinosa C.D., Abelilla J.J., Casas G.A., Lagos L.V., Lee S.A., Kwon W.B., Mathai J.K., Navarro D.M.D.L., Jaworski N.W. (2018). Non-antibiotic feed additives in diets for pigs: A review. Anim. Nutr..

[3] Windisch W., Schedle K., Plitzner C., Kroismayr A. (2008). Use of phytogenic products as feed additives for swine and poultry. J. Anim. Sci..

[4] Locatelli D.A., Nazareno M.A., Fusari C.M., Camargo A.B. (2017). Cooked garlic and antioxidant activity: Correlation with organosulfur compound composition. Food Chem..

[5] Jang H.J., Lee H.J., Yoon D.K., Ji D.S., Kim J.H., Lee C.H. (2018). Antioxidant and antimicrobial activities of fresh garlic and aged garlic by-products extracted with different solvents. Food Sci. Biotechnol..

[6] Boonpeng S., Siripongvutikorn S., Sae-wong C., Sutthirak P. (2014). The antioxidant and anti-cadmium toxicity properties of garlic extracts. Food Sci. Nutr..

[7] Li Q., Wang Y., Mai Y., Li H., Wang Z., Xu J., He X. (2020). Health Benefits of the Flavonoids from Onion: Constituents and Their Pronounced Antioxidant and Anti-neuroinflammatory Capacities. J. Agric. Food Chem..

[8] Batcioglu K., Yilmaz Z., Satilmis B., Uyumlu A.B., Erkal H.S., Yucel N., Gunal S., Serin M., Demirtas H. (2012). Investigation of in vivo radioprotective and in vitro antioxidant and antimicrobial activity of garlic (Allium Sativum). Eur. Rev. Med. Pharmacol. Sci..

[9] Vezza T., Algieri F., Garrido-Mesa J., Utrilla M.P., Rodríguez-Cabezas M.E., Baños A., Guillamón E., García F., Rodríguez-Nogales A., Gálvez J. (2019). The Immunomodulatory Properties of Propyl-Propane Thiosulfonate Contribute to its Intestinal Anti-Inflammatory Effect in Experimental Colitis. Mol. Nutr. Food Res..

[10] Naji K.M., Al-Shaibani E.S., Alhadi F.A., Al-Soudi S.A., D’souza M.R. (2017). Hepatoprotective and antioxidant effects of single clove garlic against CCl4-induced hepatic damage in rabbits. BMC Complement. Altern. Med..

[11] Moosavian S.P., Paknahad Z., Habibagahi Z. (2020). A randomized, double-blind, placebo-controlled clinical trial, evaluating the garlic supplement effects on some serum biomarkers of oxidative stress, and quality of life in women with rheumatoid arthritis. Int. J. Clin. Pract..

[12] Lee J.S., Cha Y.J., Lee K.H., Yim J.E. (2016). Onion peel extract reduces the percentage of body fat in overweight and obese subjects: A 12-week, randomized, double-blind, placebo-controlled study. Nutr. Res. Pract..

[13] Ruiz R., García M.P., Lara A., Rubio L.A. (2010). Garlic derivatives (PTS and PTS-O) differently affect the ecology of swine faecal microbiota in vitro. Vet. Microbiol..

[14] Ruiz R., Peinado M.J., Aranda-Olmedo I., Abecia L., Suárez-Pereira E., Ortiz Melle C., García Fernández J.M., Rubio L.A. (2015). Effects of feed additives on ileal mucosa-associated microbiota composition of broiler chickens. J. Anim. Sci..

[15] Peinado M.J., Ruiz R., Echávarri A., Rubio L.A. (2012). Garlic derivative propyl propane thiosulfonate is effective against broiler enteropathogens in vivo. Poult. Sci..

[16] Peinado M.J., Ruiz R., Echávarri A., Aranda-Olmedo I., Rubio L.A. (2013). Garlic derivative PTS-O modulates intestinal microbiota composition and improves digestibility in growing broiler chickens. Anim. Feed Sci. Technol..

[17] Filocamo A., Nueno-Palop C., Bisignano C., Mandalari G., Narbad A. (2012). Effect of garlic powder on the growth of commensal bacteria from the gastrointestinal tract. Phytomedicine.

[18] Quan J., Cai G., Ye J., Yang M., Ding R., Wang X., Zheng E., Fu D., Li S., Zhou S. (2018). A global comparison of the microbiome compositions of three gut locations in commercial pigs with extreme feed conversion ratios. Sci. Rep..

[19] Rubio L.A., Peinado M.J., Ruiz R., Suárez-Pereira E., Ortiz Mellet C., García Fernández J.M. (2015). Correlations between changes in intestinal microbiota composition and performance parameters in broiler chickens. J. Anim. Physiol. Anim. Nutr..

[20] McCormack U.M., Curião T., Buzoianu S.G., Prieto M.L., Ryan T., Varley P., Crispie F., Magowan E., Metzler-Zebeli B.U., Berry D. (2017). Exploring a possible link between the intestinal microbiota and feed efficiency in pigs. Appl. Environ. Microbiol..

[21] Tan Z., Yang T., Wang Y., Xing K., Zhang F., Zhao X., Ao H., Chen S., Liu J., Wang C. (2017). Metagenomic analysis of cecal microbiome identified microbiota and functional capacities associated with feed efficiency in landrace finishing pigs. Front. Microbiol..

[22] Yang H., Huang X., Fang S., He M., Zhao Y., Wu Z., Yang M., Zhang Z., Chen C., Huang L. (2017). Unraveling the fecal microbiota and metagenomic functional capacity associated with feed efficiency in pigs. Front. Microbiol..

[23] Llana-Ruiz-Cabello M., Pichardo S., Bãnos A., Núñez C., Bermúdez J.M., Guillamón E., Aucejo S., Cameán A.M. (2015). Characterisation and evaluation of PLA films containing an extract of Allium spp. to be used in the packaging of ready-to-eat salads under controlled atmospheres. LWT Food Sci. Technol..

[24] Llana-Ruiz-Cabello M., Gutiérrez-Praena D., Puerto M., Pichardo S., Moreno F.J., Baños A., Nuñez C., Guillamón E., Cameán A.M. (2015). Acute toxicological studies of the main organosulfur compound derived from Allium sp. intended to be used in active food packaging. Food Chem. Toxicol..

[25] Koné A.P., Desjardins Y., Gosselin A., Cinq-Mars D., Guay F., Saucier L. (2019). Plant extracts and essential oil product as feed additives to control rabbit meat microbial quality. Meat Sci..

[26] OECD/FAO (2019). OECD-FAO Agricultural Outlook 2019–2028.

[27] Fosse J., Seegers H., Magras C. (2008). Foodborne zoonoses due to meat: A quantitative approach for a comparative risk assessment applied to pig slaughtering in Europe. Vet. Res..

[28] De Blas C., Gasa J., Mateos G.G., López-Bote C., Gorrachategui M., Aguilera J., Fructuoso G. (2013). Necesidades Nutricionales Para Ganado Porcino Normas FEDNA.

[29] Tecles F., Rubio C.P., Contreras-Aguilar M.D., López-Arjona M., Martínez-Miró S., Martínez-Subiela S., Cerón J.J. (2018). Adenosine deaminase activity in pig saliva: Analytical validation of two spectrophotometric assays. J. Vet. Diagn. Investig..

[30] AOAC (2006). Official Methods of Analysis of the Association Official Analytical Chemists.

[31] Van Soest P.J., Robertson J.B., Lewis B.A. (1991). Methods for Dietary Fiber, Neutral Detergent Fiber, and Nonstarch Polysaccharides in Relation to Animal Nutrition. J. Dairy Sci..

[32] Boletín Oficial del Estado (2000). Métodos Oficiales de análisis de piensos o alimentos para animales y sus primeras materias. BOE.

[33] Abad P., Arroyo-Manzanares N., García-Campaña A.M. (2016). A rapid and simple UHPLC-ESI-MS/MS method for the screening of propyl propane thiosulfonate, a new additive for animal feed. Anal. Methods.

[34] Rubio C.P., Mainau E., Cerón J.J., Contreras-Aguilar M.D., Martínez-Subiela S., Navarro E., Tecles F., Manteca X., Escribano D. (2019). Biomarkers of oxidative stress in saliva in pigs: Analytical validation and changes in lactation. BMC Vet. Res..

[35] Oliveira L., Madrid J., Ramis G., Martínez S., Orengo J., Villodre C., Valera L., López M.J., Pallarés F.J., Quereda J.J. (2014). Adding crude glycerin to nursery pig diet: Effect on nutrient digestibility, metabolic status, intestinal morphology and intestinal cytokine expression. Livest. Sci..

[36] Leser T.D., Amenuvor J.Z., Jensen T.K., Lindecrona R.H., Boye M., Moøller K. (2002). Culture-independent analysis of gut bacteria: The pig gastrointestinal tract microbiota revisited. Appl. Environ. Microbiol..

[37] Sghir A., Gramet G., Suau A., Rochet V., Pochart P., Dore J. (2000). Quantification of bacterial groups within human fecal flora by oligonucleotide probe hybridization. Appl. Environ. Microbiol..

[38] Penders J., Thijs C., Van Den Brandt P.A., Kummeling I., Snijders B., Stelma F., Adams H., Van Ree R., Stobberingh E.E. (2007). Gut microbiota composition and development of atopic manifestations in infancy: The KOALA birth cohort study. Gut.

[39] Delroisse J.M., Boulvin A.L., Parmentier I., Dauphin R.D., Vandenbol M., Portetelle D. (2008). Quantification of *Bifidobacterium* spp. and *Lactobacillus* spp. in rat fecal samples by real-time PCR. Microbiol. Res..

[40] Revolledo L., Ferreira A.J.P. (2010). Salmonella antibiotic-mutant strains reduce fecal shedding and organ invasion in broiler chicks. Poult. Sci..

[41] Rinttilä T., Kassinen A., Malinen E., Krogius L., Palva A. (2004). Development of an extensive set of 16S rDNA-targeted primers for quantification of pathogenic and indigenous bacteria in faecal samples by real-time PCR. J. Appl. Microbiol..

[42] Castillo M., Martín-Orúe S.M., Manzanilla E.G., Badiola I., Martín M., Gasa J. (2006). Quantification of total bacteria, enterobacteria and lactobacilli populations in pig digesta by real-time PCR. Vet. Microbiol..

[43] Samolińska W., Grela E.R., Kowalczuk-Vasilev E., Kiczorowska B., Klebaniuk R., Hanczakowska E. (2020). Evaluation of garlic and dandelion supplementation on the growth performance, carcass traits, and fatty acid composition of growing-finishing pigs. Anim. Feed Sci. Technol..

[44] Cullen S.P., Monahan F.J., Callan J.J., O’Doherty J.V. (2005). The effect of dietary garlic and rosemary on grower-finisher pig performance and sensory characteristics of pork. Irish J. Agric. Food Res..

[45] Ramakrishna Rao R., Platel K., Srinivasan K. (2003). In vitro influence of spices and spice-active principles on digestive enzymes of rat pancreas and small intestine. Nahr. Food.

[46] Kothari D., Do Lee W., Niu K.M., Kim S.K. (2019). The genus Allium as poultry feed additive: A review. Animals.

[47] Mellor S. (2000). Herbs and spices promote health and growth. Pig Prog..

[48] Omojola A., Fagbuaro S., Ayeni A. (2009). Cholesterol content, physical and sensory properties of pork from pigs fed varying levels of dietary garlic (*Allium sativum*). World Appl. Sci. J..

[49] Grela E.R., Pietrzak K., Sobolewska S., Witkowski P. (2013). Efektywność dodatku inuliny i czosnku w żywieniu tuczników. Ann. Anim. Sci..

[50] Batiha G.E.S., Beshbishy A.M., Wasef L.G., Elewa Y.H.A., Al-Sagan A.A., El-Hack M.E.A., Taha A.E., Abd-Elhakim Y.M., Devkota H.P. (2020). Chemical constituents and pharmacological activities of garlic (*Allium sativum* L.): A review. Nutrients.

[51] Focke M., Feld A., Lichtenthaler H.K. (1990). Allicin, a naturally occurring antibiotic from garlic, specifically inhibits acetyl-CoA synthetase. FEBS Lett..

[52] Rees L.P., Minney S.F., Plummer N.T., Slater J.H., Skyrme D.A. (1993). A quantitative assessment of the antimicrobial activity of garlic (*Allium sativum*). World J. Microbiol. Biotechnol..

[53] Miron T., Rabinkov A., Mirelman D., Wilchek M., Weiner L. (2000). The mode of action of allicin: Its ready permeability through phospholipid membranes may contribute to its biological activity. Biochim. Biophys. Acta Biomembr..

[54] Sorlozano-Puerto A., Albertuz-Crespo M., Lopez-Machado I., Ariza-Romero J.J., Baños-Arjona A., Exposito-Ruiz M., Gutierrez-Fernandez J. (2018). In vitro antibacterial activity of propyl-propane-thiosulfinate and propyl-propane-thiosulfonate derived from allium spp. Against gram-negative and gram-positive multidrug-resistant bacteria isolated from human samples. Biomed. Res. Int..

[55] Aumiller T., Mosenthin R., Weiss E. (2015). Potential of cereal grains and grain legumes in modulating pigs’ intestinal microbiota—A review. Livest. Sci..

[56] He T., Zhu Y.H., Yu J., Xia B., Liu X., Yang G.Y., Su J.H., Guo L., Wang M.L., Wang J.F. (2019). Lactobacillus johnsonii L531 reduces pathogen load and helps maintain short-chain fatty acid levels in the intestines of pigs challenged with Salmonella enterica Infantis. Vet. Microbiol..

[57] Macfarlane S., Macfarlane G.T. (2003). Regulation of short-chain fatty acid production. Proc. Nutr. Soc..

[58] Meimandipour A., Hair-Bejo M., Shuhaimi M., Azhar K., Soleimani A.F., Rasti B., Yazid A.M. (2010). Gastrointestinal tract morphological alteration by unpleasant physical treatment and modulating role of Lactobacillus in broilers. Br. Poult. Sci..

[59] Macfarlane G.T., Gibson G.R., Beatty E., Cummings J.H. (1992). Estimation of short-chain fatty acid production from protein by human intestinal bacteria based on branched-chain fatty acid measurements. FEMS Microbiol. Lett..

[60] Smith E.A., MacFarlane G.T. (1998). Enumeration of amino acid fermenting bacteria in the human large intestine: Effects of pH and starch on peptide metabolism and dissimilation of amino acids. FEMS Microbiol. Ecol..

[61] Leong J., Morel P.C.H., Purchas R.W., Wilkinson B.H.P. (2011). Effects of dietary components including garlic on concentrations of skatole and indole in subcutaneous fat of female pigs. Meat Sci..

[62] Banerjee S.K., Maulik M., Manchanda S.C., Dinda A.K., Das T.K., Maulik S.K. (2001). Garlic-induced alteration in rat liver and kidney morphology and associated changes in endogenous antioxidant status. Food Chem. Toxicol..

[63] Gorinstein S., Leontowicz M., Leontowicz H., Najman K., Namiesnik J., Park Y.S., Jung S.T., Kang S.G., Trakhtenberg S. (2006). Supplementation of garlic lowers lipids and increases antioxidant capacity in plasma of rats. Nutr. Res..

